# Incidence of infection associated with eculizumab: a meta-analysis of 9 randomized controlled trials

**DOI:** 10.3389/fphar.2025.1538563

**Published:** 2025-04-28

**Authors:** Aidou Jiang, Ying Liu, Chunyan Wei, Guirong Xiao

**Affiliations:** Department of Pharmacy, West China Hospital, Sichuan University, Chengdu, China

**Keywords:** eculizumab, infection, bacteremia, meta-analysis, urinary tract infection

## Abstract

**Background and Aims:**

Eculizumab is expected to lead to increased susceptibility to infection. We performed a meta-analysis of data from randomized controlled trials (RCTs) to determine the risk of infection in eculizumab-treated patients.

**Methods:**

We searched PubMed, EMBASE, Web of Science and ClinicalTrials.gov (up to 8 Oct 2024) to identify published RCTs that focused on the occurrence of infection in patients treated with eculizumab regardless of the indications of the patients. Relative risks and 95% confidence intervals (95% CIs) were calculated via the random effects model. (PROSPERO Code No. CRD42024562470).

**Results:**

Nine RCTs including 691 patients were eligible. Compared with the control (placebo or standard of care), eculizumab did not significantly increase the overall risk of infection (RR = 1.07; 95% CI, 0.89–1.28; *I* ^2^ = 44%), regardless of whether the infection was a general infection (RR = 1.07; 95% CI, 0.86–1.34; *I* ^2^ = 39%) or a serious infection (RR = 1.05; 95% CI, 0.75–1.47; *I* ^2^ = 11%). Analyses of subgroups revealed that eculizumab significantly increased the risk of general urinary system infection (RR = 1.33; 95% CI, 1.00–1.77; *I* ^2^ = 46%) and severe bacteremia (RR = 2.31; 95% CI, 1.04–5.13; *I* ^2^ = 0%).

**Conclusion:**

Compared with placebo or standard of care, although eculizumab did not significantly increase the overall risk of infection, it was associated with 33% and 131% increases in the risk of general urinary system infection and severe bacteremia, respectively.

**Systematic Review registration:**

PROSPERO CRD42024562470

## Introduction

Eculizumab is a humanized monoclonal antibody to complement factor 5 that blocks complement activation. It was first used to prevent the breakdown of red blood cells in adults with paroxysmal nocturnal hemoglobinuria (PNH) and was approved by the United States Food and Drug Administration (FDA) in 2007. It is also used in adults and children weighing at least 5 kg to treat a blood disease called atypical hemolytic uremia (aHUS, approved in 2011), as well as generalized myasthenia gravis (gMG, approved in 2017) with positive anti-acetylcholine receptor (AchR) and neuromyelitis optica spectrum disorder with positive anti-aquaporin-4 (AQP4) (NMOSD, approved in 2019) (Patriquin and Kuo, 2019; Zhang et al., 2024). On 28 May 2024, the FDA approved eculizumab-aeeb as the first interchangeable biosimilar to eculizumab. One month later, on 22 July 2024, the FDA approved eculizumab-aagh. Both are approved as interchangeable biosimilars to eculizumab (brand name: Soliris) to treat the following indications: aHUS and PNH.

In recent years, there has been a high demand for the use of eculizumab in an increasing number of diseases characterized by complement dysregulation, whether it is on-label or off-label. The FDA has added a black box warning regarding serious and life-threatening infections caused by *Neisseria meningitidis* (Avasarala et al., 2021). Eculizumab specifically binds to the complement protein C5 with high affinity, thereby inhibiting its cleavage to C5a and C5b and preventing the generation of the terminal complement complex C5b-9 (Patriquin and Kuo, 2019). For these reasons, eculizumab indeed increases the infection risk of capsule-forming bacteria such as *Neisseria* spp. (Niitsuma-Sugaya et al., 2021). In addition to *Neisseria* spp., severe *Neisseria* gonorrheae (Niitsuma-Sugaya et al., 2021), bacteremia (Kawakami et al., 2018), Cryptococcus (Lortholary et al., 2023) and virus-related infections (Okusa et al., 2024) have been reported.

As eculizumab is expected to lead to increased susceptibility to infection, more studies on the incidence of infection induced by eculizumab are needed. To the best of our knowledge, no studies on this incidence have been published in clinical trials. We therefore summarized all available evidence from RCTs for a comprehensive and rigorous meta-analysis of the risk of infection associated with eculizumab.

## Methods

### Data sources and searches

We followed the Preferred Reporting Items for Systematic Reviews and Meta-Analyses (PRISMA) statement for reporting systematic reviews (Liberati et al., 2009) and the standards of the Cochrane Collaboration. We searched PubMed, EMBASE, Web of Science and ClinicalTrials.gov (up to 8 Oct 2024) to identify published RCTs that focused on patients treated with eculizumab regardless of their indications. The search terms used were as follows: (“eculizumab” OR “5G1.1” OR “H5G1.1VHC + H5G1.1VLC” OR “h5g1 1” OR “Elizaria” OR “Soliris” OR “Alexion”) AND (“clinical trial” OR “controlled clinical trial” OR “randomized controlled trials” OR “intervention study” OR “clinical trials randomized” OR “trials randomized clinical” OR “controlled clinical trials randomized”). The complete search strategy can be found in Supplementary Table S1.

### Eligibility criteria and outcomes

Studies were considered for inclusion if they met the following criteria: (1) they were RCTs reported in full-text publications; single-arm clinical trials were excluded from the study; (2) they used eculizumab treatment as the experimental drug; (3) they used placebo or standard treatment as controls, and standard treatment was the standard of care or care as usual; and (4) the infection was reported as an adverse event. The protocol (PROSPERO Code No. CRD42024562470) was submitted to the International Prospective Register of Systematic Reviews. Two reviewers (AJ and YL) independently screened all the citations from the initial search. Any discrepancy was referred to a third reviewer (GX) and resolved by discussion.

The primary outcome of this study was overall infection, and the secondary outcomes were general infection, serious infection, and different types of infection. According to the definition of serious adverse events in clinical studies on the clinicaltrials.gov website, serious infection was defined as an adverse event with the following results: (1) life-threatening or resulting in death and (2) patient hospitalization or extension of a current hospital stay, resulting in an ongoing or significant incapacity for or interference with normal life functions. The others were considered to have a general infection.

### Data extraction

Two trained investigators (AJ and YL) independently extracted the data via a predefined data extraction form, which included the first author’s name, year of publication, sample size, study design (intervention groups and control groups), duration of follow-up, country of origin, patient characteristics (age and sex), receipt of the meningococcal vaccine, use of eculizumab, dose of eculizumab, and data concerning infection events. Published data or posted results on the clinicaltrials.gov platform were collected for each of the studies, which included upper respiratory tract infection (nasopharyngitis, sinusitis, etc.), lower respiratory tract or lung infection (bronchitis, pneumonia, etc.), virus infection, digestive system infection (appendicitis, gastroenteritis, diverticulitis, etc.), urinary system infection (urinary tract infection, pyelonephritis, etc.), bacteremia and other infections such as cellulitis, abscess and so on.

### Quality evaluation

The methodological quality of each included RCT was assessed according to the Cochrane Collaboration Risk of Bias Tool (Savović et al., 2014). The quality of the trials was judged as low, unclear, or high in terms of the risk of bias on the basis of the following domains: random sequence generation (selection bias), allocation concealment (selection bias), blinding (performance bias and detection bias), incomplete outcome data (attrition bias), and selective reporting (reporting bias).

### Statistical analysis

A meta-analysis was performed with the software Review Manager 5.4 (Nordic Cochrane Centre, The Cochrane Collaboration). Relative risks (RRs) and their 95% confidence intervals (95% CIs) were used to calculate the comparative effect sizes, with *P* < 0.05 indicating a statistically significant difference. The heterogeneity between studies was examined via the Q statistic test and the *I* ^2^ test. No statistical heterogeneity was deemed to exist if *P* > 0.05 and *I* ^2^ 50% were present. The fixed effects model was applied if there was no statistical heterogeneity between studies. Otherwise, the random effects model was applied. Subgroup analyses were performed according to the severity of infection (general infection or severe infection) and different types of infection events. Potential publication bias was evaluated by visually inspecting the funnel plots (Higgins et al., 2003).

## Results

### Study selection

**FIGURE 1:**
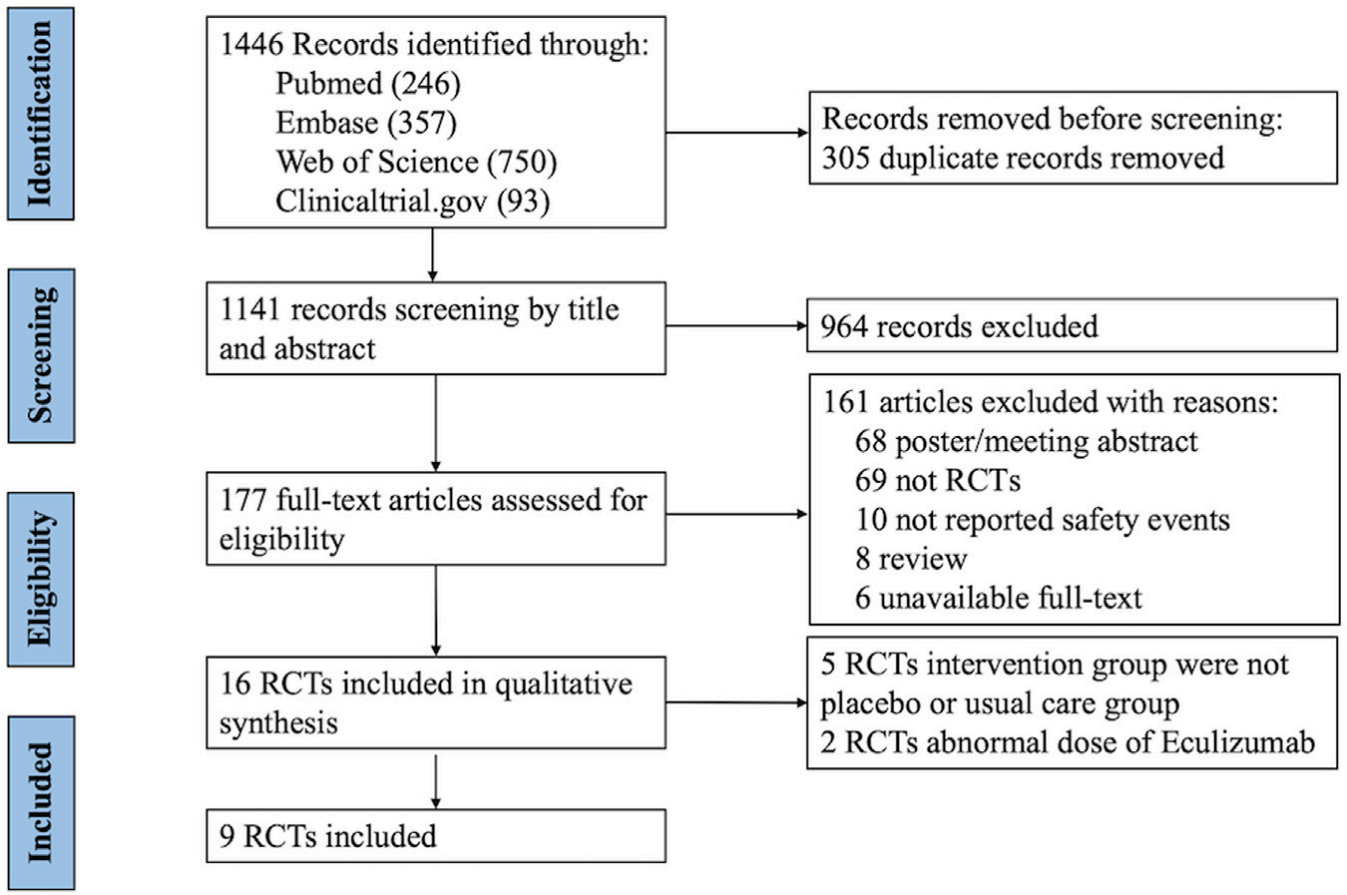
PRISMA (Preferred Reporting Items for Systematic Reviews and Meta-Analyses) flow diagram: randomized controlled trials included and excluded from this meta-analysis. RCTs, randomized controlled trials.

Initially, 1,446 studies were identified from the selected databases, and after the removal of duplicates, 1,141 studies remained. After the titles and abstracts were screened, only 177 studies were included in the full-text screening process. Finally, nine randomized controlled trials (RCTs) (Garnier et al., 2023; Kuwabara et al., 2024; Hillmen et al., 2006; Howard et al., 2013; 2017; Kulkarni et al., 2017; Misawa et al., 2018; Marks et al., 2019; Pittock et al., 2019) were included in this meta-analysis. The study screening process and the results are shown in Figure 1.

### Study characteristics

A total of 691 participants from nine RCTs were included in the study. A total of 386 of these patients were assigned to eculizumab treatment plans. The characteristics of the included RCTs are summarized in Table 1. On the clinical trial registration website, the protocols for nine trials have been posted publicly. Details of the quality evaluation are summarized in Supplementary Table S2. Among the nine RCTs, three (Kulkarni et al., 2017; Marks et al., 2019; Garnier et al., 2023) were non-double-blind clinical studies; therefore, we considered the quality of the evidence to be moderate. Details of the quality evaluation are summarized in Supplementary Figure S1.

**TABLE 1:** Demographic and clinical characteristics of the included studies.

Article	Clinical trials registration	Center	Indication	Sample	Age of patients	Intervention group				Control group		RCT duration	Follow-up
						n	Treatment	Vaccinated against *Neisseria* meningitides	Antibiotic prophylaxis	n	Treatment		
Hillmen et al. (2006)	NCT00122330	Muti-center	PNH	87	Adult	43	Eculizumab 600 mg weekly for 4 weeks, then 900 mg every 2 weeks	Yes	-	44	Placebo	26 weeks	26 weeks
Howard et al. (2013)	NCT00727194	Single-center (America)	severe, refractory gMG	14	Adult	14	Eculizumab 600 mg weekly for 4 weeks, then 900 mg every 2 weeks	Yes	-	14	Placebo	16 weeks	46 weeks
Howard et al. (2017)	NCT01997229	Muti-center	AchR positive refractory gMG	125	Adult	62	Eculizumab 900 mg on day 1 and weeks 1, 2, and 3; 1,200 mg at week 4; and 1,200 mg every 2 weeks	Yes	-	63	Placebo	26 weeks	34 weeks
Kulkarni et al. (2017)	NCT01327573	Single-center (America)	Chronic Antibody-Mediated Injury in Kidney Transplant Recipients	15	Adult	10	Eculizumab 600 mg weekly for 4 weeks, then 900 mg every 2 weeks	Yes	-	5	Placebo	26 weeks	52 weeks
Misawa et al. (2018)	NCT02493725	Single-center (Japan)	Guillain‒Barré syndrome	34	Adult	23	Eculizumab 900 mg weekly for a total of 4 doses (days 1, 8, 15, and 22) plus IVIg of 400 mg/kg once daily for 5 days	Not required	Yes, up to 8 weeks	11	Placebo	4 weeks	24 weeks
Kuwabara et al. (2024)	NCT04752566	Single-center (Japan)	Guillain‒Barré syndrome	57	Adult	37	Eculizumab 900 mg weekly for a total of 4 doses (days 1, 8, 15, and 22) plus IVIg of 400 mg/kg once daily for 5 days	Not required	Yes, up to 8 weeks	20	Placebo	4 weeks	24 weeks
Marks et al. (2019)	NCT01399593	Muti-center	prevent antibody-mediated rejection in living-donor kidney transplant recipients	102	Adult	51	Eculizumab 1,200 mg on day 0, 900 mg on day 1 and weeks 1, 2, 3 and 4; 1,200 mg at week 5, 7, 9	Yes	Yes, according to local antibiotic practice	51	Standard of care	9 weeks	3 years
Pittock et al. (2019)	NCT01892345	Muti-center	NMOSD	143	Adult	96	Eculizumab 900 mg weekly for 4 weeks, then 1,200 mg every 2 weeks until relapse, trial discontinuation, or the end of the trial	Yes	-	47	Placebo	211 weeks	211 weeks
Garnier et al. (2023)	NCT02205541	Single-center (France)	Shiga Toxin–Related HUS	100	Pediatric	50	Eculizumab on day 0, 7, 14, 21, and 28, doses according to the bodyweight	Yes	Yes, oral penicillin, up to 60 days after last injection	50	Placebo	4 weeks	52 weeks

### Overall risk of infection

**FIGURE 2:**
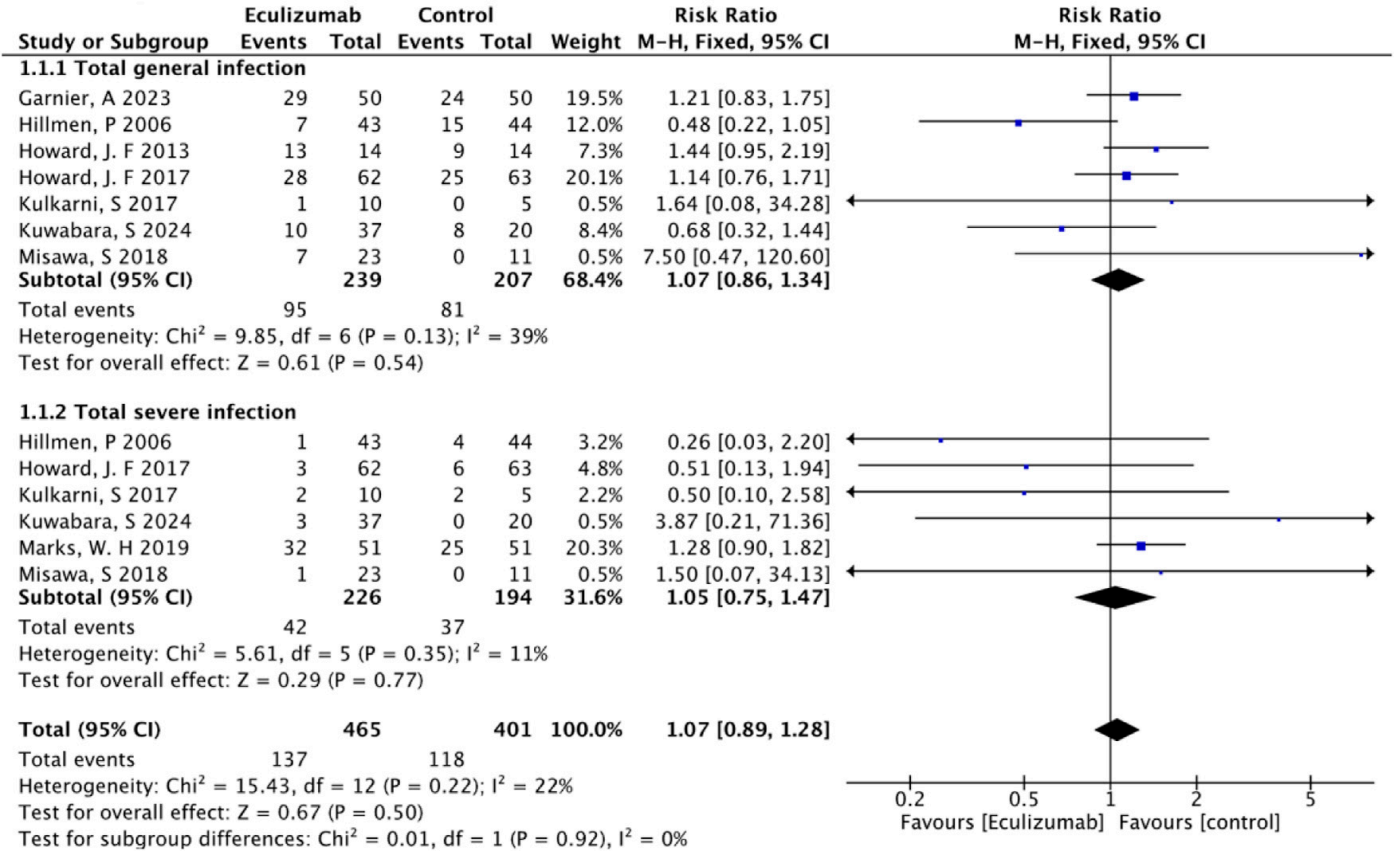
Forest plot with meta-analysis of the overall risk of infection.

The overall rate of infection was 46.53% (255/548) after pooling the data from the 8 RCTs (excluding Pittock, S. J.'s study (Pittock et al., 2019), owing to the lack of total number of infected patients): 47.24% (137/290) in the eculizumab-treated group and 45.73% (118/258) in the control group. Compared with the control, eculizumab insignificantly increased the overall risk of infection (RR = 1.07; 95% CI, 0.89–1.28; *I* ^2^ = 44%). The subgroup analysis indicated that both general infection (RR = 1.07; 95% CI, 0.86–1.34; *I* ^2^ = 39%) and serious infection (RR = 1.05; 95% CI, 0.75–1.47; *I* ^2^ = 11%) were not significantly different between the eculizumab-treated group and the control group (Figure 2).

### Risk of infection by different follow-ups

Due to heterogeneity in follow-up durations across studies, we performed subgroup analyses stratified by different follow-up periods (<26 weeks, 26–52 weeks, and >52 weeks). For total general infection, data limitations permitted analysis only for < 26 weeks and 26–52 weeks subgroups. In the 26–52 weeks subgroup, eculizumab appeared to exhibit a higher risk of general infection (RR = 1.22; 95% CI: 0.96–1.55; *I* ^2^ = 0%), though this difference did not reach statistical significance (Supplementary Figure S3). In contrast, no similar trend was observed for total severe infection. While the >52 weeks follow-up subgroup suggested a potential increase in infection risk (RR = 1.28; 95% CI: 0.90–1.82, *I* ^2^ = 0%), interpretation is limited by the inclusion of only a single RCT, warranting further investigation (Supplementary Figure S4).

### Risk of infection by type of infection

As shown in Table 2, subgroup analyses were conducted for different infection types. Compared with the control, eculizumab significantly increased the risk of general urinary system infection (RR = 1.33; 95% CI, 1.00–1.77; *I* ^2^ = 46%) and severe bacteremia (RR = 2.31; 95% CI, 1.04–5.13; *I* ^2^ = 0%). Infections such as cellulitis, conjunctivitis, and cryptococcal infection, which cannot be classified according to the upper or lower respiratory tract, urinary system or other systems, were unified into other infections. The difference in the risk of severe other infections (RR = 2.27; 95% CI, 0.98–5.24; *I* ^2^ = 0%) between the eculizumab group and the control group nearly matched the significance standard, but *P* = 0.06. No significant difference in the risk of other types of infection was found.

**TABLE 2:** Subgroup analysis of the included published studies.

Type of infection	Number of studies	Sample size	Risk Ratio	95% CI	P
General upper respiratory	7	572	1.20	0.81–1.76	0.36
Severe upper respiratory	4	457	0.25	0.06–1.03	0.05
General lower respiratory	5	383	0.72	0.41–1.26	0.25
Severe lower respiratory	3	300	1.46	0.62–3.42	0.38
General digestive system infection	2	157	1.94	0.76–4.93	0.17
Severe digestive system infection	3	370	0.35	0.11–1.12	0.08
General urinary system infection	5	482	1.33	1.00–1.77	0.05
Severe urinary system infection	4	457	0.90	0.54–1.49	0.68
Severe bacteremia	4	457	2.31	1.04–5.13	0.04
General virus infection	7	467	1.21	0.84–1.75	0.31
Severe virus infection	6	529	0.76	0.28–2.10	0.60
General other infection	6	417	1.25	0.79–1.98	0.34
Severe other infection	6	506	2.27	0.98–5.24	0.06

### Sensitivity analyses

In this meta-analysis, studies that used an active medicine as the control group (pevacizumab, ravulizumab, or eculizumab biosimilar) or whose follow-up durations were < 20 weeks were excluded. The leave-1-out sensitivity analysis failed to identify any individual trial as having influenced the primary outcome. These findings confirmed the robustness of the primary results (Supplementary Table S3).

### Publication bias

The results showed that the funnel plot was asymmetrical, potentially indicating publication bias owing to the small sample size of the included studies and the lack of statistical significance (Supplementary Figure S2).

## Discussion

As eculizumab increases susceptibility to infection, along with its high efficacy, it has been widely used for different indications and for different diseases. However, whether eculizumab increases the risk of infection remains uncertain. This meta-analysis is the first to provide a comprehensive overview of eculizumab-associated infection risk on the basis of nine RCTs, including 691 patients. The major findings were as follows: (1) eculizumab did not increase the risk of overall infection or the incidence of both general and serious infections; (2) eculizumab use was associated with a greater risk of general urinary system infection and severe bacteraemia; and (3) eculizumab use might increase the risk of other severe infections, such as cellulitis and conjunctivitis.

Several meta-analyses and systematic reviews have focused on eculizumab. However, owing to its large-scale application and indications in different systemic diseases, these meta-analyses are based on the efficacy or safety of one disease (Beyer and Fridovich, 1987; Zhou et al., 2021; Tang et al., 2023). In these studies, the overall incidence of adverse events (AEs) was considered, but no systematic analysis of specific adverse events (such as infection) was performed. However, eculizumab-induced infections were previously limited to those reported on *N. meningitidis* and *Neisseria* gonorrheae infections. In clinical treatment, eculizumab is contraindicated in patients who are not currently vaccinated against *N. meningitidis* unless the risk of delaying eculizumab treatment outweighs the risk of developing a meningococcal infection. Seven of nine included RCTs required patients to have been vaccinated against *Neisseria* meningitides. In the other two studies, if patients’ meningococcal vaccination was not performed, all enrolled patients received antibiotic prophylaxis against *N*. *meningitidis* from the time of the first dose of the study drug to 8 weeks after the last administration. Currently, several pharmacovigilance studies have investigated the FAERS database and published literature on eculizumab-related infection adverse events (Okusa et al., 2024; Zhang et al., 2024). Pharmacovigilance studies have shown that eculizumab is associated with a significantly increased risk of *N. meningitidis* infection, as well as gonococcal and streptococcal infection. Considering an important limitation of that study, i.e., that only some AEs with a high incidence were retrieved, their assessment of the risk of infection was not comprehensive. The Associazione Italiana Emoglobinuria Parossistica Notturna (AIEPN) (Girmenia et al., 2023) has produced recommendations for the management of infection in PNH patients treated with eculizumab, but this recommendation is still limited to *Neisseria* spp. Therefore, considering the limitations of previous studies, this meta-analysis included all the infection events reported in RCTs, regardless of whether they were common or not, to systematically evaluate the risk of infection associated with eculizumab. Eculizumab increases the risk of life-threatening infections with *Neisseria* spp. The estimated risk is 0.5% per year or 5% after 10 years of treatment (Goh et al., 2024). The absence of *Neisseria* infections in our analysis may be attributed to effective vaccination protocols in most studies, comprehensive antibiotic prophylaxis strategies and potential sample size and the duration of follow-up limitations. However, the potential risk should not be underestimated. This underscores the importance of sustained vigilance and patient education. However, the risk of *Neisseria* infection should be closely monitored in clinical practice because vaccinated patients also have the possibility of becoming infected (McNamara et al., 2017). With respect to *Neisseria* gonorrheae infections, guidelines do not suggest any prevention strategy other than paying attention to lifestyle (Girmenia et al., 2023).

Moreover, in addition to the currently published study data, we also included data on infection-related adverse effects published publicly available on the ClinicalTrials.gov website to make the study more comprehensive. We ultimately confirmed that, compared with placebo or standard of care, eculizumab was associated with a relatively high risk of severe bacteremia and other infections. However, three RCTs in the article did not adopt a double-blind design, which may introduce bias. The diagnosis of bacteremia primarily relies on laboratory tests (e.g., blood cultures), which are less susceptible to subjective influence and thus may have lower bias. In contrast, the diagnosis of urinary tract infection (UTI) depends not only on urine cultures but may also involve symptom reporting (e.g., urinary frequency, dysuria), making it more prone to reporting bias in non-blinded trials, potentially leading to false-positive results. We acknowledge that the impact of underreporting mild infections cannot be entirely ruled out, which may lead to an underestimation of the overall infection risk and an overestimation of the relative risk of severe infections. Nevertheless, considering all the evidence, we believe the main conclusions remain valid.

Eculizumab has diverse clinical indications, and many of the included RCTs investigated off-label uses of this drug. Notably, we observed variations in the initial dosing regimens of eculizumab across studies (600 mg weekly vs. 900 mg weekly). 3/4 studies reporting severe bacteremia used 900mg, suggesting dose-related risk - though confirmation is limited by scarce 600 mg data (Supplementary Figure S5). However, this observation requires cautious interpretation due to the limited number of RCTs using the 600 mg regimen. Further studies are needed to validate this finding.

We also assessed the association of bacteremia with prophylactic antibiotic use during eculizumab treatment. However, of the four RCTs included, three studies did not use antibiotics prophylactically, whereas only one RCT was used; thus, more studies are needed in the future.

In view of related studies (Marks et al., 2019; Pittock et al., 2019), the high incidence of general urinary tract infections (including urinary tract infections, cystitis, pyelonephritis, etc.) in patients treated with eculizumab, especially in patients with underlying kidney diseases, requires special attention. In Garnier et al. (Garnier et al., 2023) and Marks et al. (Marks et al., 2019)studies, patients had higher baseline creatinine levels, and most patients were already on dialysis when receiving eculizumab treatment. Structural urinary tract abnormalities, voiding dysfunction, or concomitant use of immunosuppressants in patients with underlying kidney diseases may have potentially influenced the results. Underlying kidney disease may increase susceptibility to UTIs, as several studies related to complement inhibitors have reported corresponding UTI risks (Gäckler et al., 2021; Socié et al., 2019). Therefore, in clinical practice, it is recommended to strengthen UTI monitoring in patients receiving complement inhibitors, especially those with renal insufficiency. For example, clinicians should first conduct a comprehensive assessment of patients’ conditions for possible risk factors, such as age, history of infection, history of urinary surgery, and history of immunosuppressant exposure and vaccination.

Second, this study suggests a high risk of severe bacteremia associated with eculizumab (with or without antibiotic prophylaxis). Therefore, prophylaxis of meningococcal infections with antibiotics should be given until 14 days after the administration of the meningococcal vaccine. Alternatively, long-term antibiotic prophylaxis has been implemented in some countries. While waiting for more data, a cautious approach is advised. Third, in our findings, the incidence of other serious infections (including cellulitis, otitis media, conjunctivitis, or some fungal-related infections that do not indicate the site of infection) was higher in the eculizumab group than in the placebo group, but the difference was not significant. However, adequate laboratory and instrumental tests are also necessary to make an early diagnosis and promptly start treatment where appropriate. Current guidelines (Lee et al., 2020; Girmenia et al., 2023; Goh et al., 2024; Red Blood Cell Disease GroupChinese Society of HematologyChinese Medical Association, 2024) primarily emphasize meningococcal infection prophylaxis, while few mentioned routine infection monitoring. Notably, no specific recommendations address UTI/bacteremia surveillance. Based on our risk stratification analysis, we propose the following evidence-based recommendations: (1) inclusion of severe bacteremia in eculizumab’s significant adverse drug reaction profile; (2) development of tailored monitoring algorithms for high-risk populations. For all eculizumab-treated patients, immediately take the blood cultures during febrile episodes. High-risk subgroups (e.g., indwelling catheters) could consider periodic inflammatory marker testing (e.g., C-reactive protein). Urine cultures for all symptomatic UTIs in eculizumab-treated patients; (3) implementation of targeted clinical trials to assess active surveillance strategies. It should be noted that the inherent limitations of meta-analytic methodology preclude definitive conclusions regarding optimal screening frequency, which would require prospective cohort studies. In summary, although the overall risk of eculizumab infection is not clearly elevated, it is important to understand and identify the risk of bacteremia or urinary tract infection with eculizumab as early as possible for clinical treatment.

## Limitations

The major advantage of this study was that we comprehensively assessed the risk of infection in patients treated with eculizumab on the basis of evidence from RCTs. There are several limitations in this meta-analysis. First, three of the included RCTs were non-double-blind studies, with moderate evidence of quality, although the sensitivity analysis revealed that their effect on the results was not significant. Second, the clinical trials included in our study were performed at various international institutions, which might have varying expertise and ability to detect infection, making it possible that the reported incidence was biased. In addition, this meta-analysis included all indications for eculizumab, and some inherent differences in the underlying conditions of patients may have affected the results. Fourth, the timing of infection occurrence might be related to the duration of treatment and follow-up. We initially excluded patients with less than 4 weeks of treatment, but the longer the follow-up period was, the greater the number of reported infections was; therefore, the results must be interpreted with caution. Fifth, owing to the limited number of cases, certain infections (otitis media acute, tinea pedis, cellulitis, etc.) were not included in the subgroup of different types of infection in the meta-analysis. Finally, this study only evaluated the infection risk of eculizumab on the basis of data from RCTs; to extend RCT findings to large patient populations in real-world clinical practice, further research with larger samples of real-world studies evaluating eculizumab safety is necessary.

## Conclusion

In conclusion, by systematically evaluating evidence from RCTs, we confirmed that although eculizumab did not significantly increase the overall risk of infection, regardless of general or severe infection, it did significantly increase the risk of general urinary system infection and severe bacteremia. These findings can help clinicians assess the risk of infection in patients treated with eculizumab.

## Data Availability

The original contributions presented in the study are included in the article/Supplementary Material, further inquiries can be directed to the corresponding author.
