# Supplementary material for: Incidence of infection associated with eculizumab: a meta-analysis of 9 randomized controlled trials
Source: Front Pharmacol. 2025 Apr 28;16:1538563. doi: 10.3389/fphar.2025.1538563 (PMC12067414; doi:10.3389/fphar.2025.1538563)
Supplement: Supplementary file 1 [file DataSheet1.docx]

Supplementary Material

**Incidence of infection associated with eculizumab: a meta-analysis of 9 randomized controlled trials**

**Supplementary Table S1. Search strategies for the meta-analysis.**

|  | Terms | Number |
| --- | --- | --- |
| ***PubMed*** | | |
|  | ("clinical trial"[Text Word] OR "controlled clinical trial"[Text Word] OR "randomized controlled trials"[Text Word] OR "intervention study"[Text Word] OR "clinical trials randomized"[Text Word] OR "trials randomized clinical"[Text Word] OR "controlled clinical trials randomized"[Text Word]) AND ("eculizumab"[Supplementary Concept] OR "eculizumab"[Title/Abstract] OR "5G1.1"[Title/Abstract] OR "H5G1.1VHC+H5G1.1VLC"[Title/Abstract] OR "h5g1 1"[Title/Abstract] OR "h5g1 1"[Title/Abstract] OR "Elizaria"[Title/Abstract] OR "Soliris"[Title/Abstract] OR "Alexion"[Title/Abstract]) | 246 |
| Embase | | |
|  | 'eculizumab'/exp OR 'eculizumab') AND ('clinical trial':ab OR 'controlled clinical trial':ab OR 'randomized controlled rials':ab). | 357 |
| Web of science | | |
|  | (AB=( eculizumab OR Elizaria OR Soliris OR Alexion) AND (AB=( (clinical trial OR controlled clinical trial OR randomized controlled trials OR intervention study OR clinical trials randomized OR trials randomized clinical OR controlled clinical trials randomized) OR TS=(clinical trial OR controlled clinical trial OR randomized controlled trials OR intervention study OR clinical trials randomized OR trials randomized clinical OR controlled clinical trials randomized) ) | 750 |
| Clinicaltrials.gov | | |
|  | Eculizumab \| Interventional studies | 93 |

**Supplementary Table S2. Sensitivity analyses of the risk of infection** **associated with Eculizumab.**

| **Study Omitted** | **RR** | **95%CI** |
| --- | --- | --- |
| Hillmen, P 2006 | 1.19 | 0.99-1.43 |
| Howard, J. F 2013 | 1.04 | 0.86-1.02 |
| Howard, J. F 2017 | 1.09 | 0.89-1.34 |
| Kulkarni, S 2017 | 1.08 | 0.90-1.29 |
| Misawa, S 2018 | 1.03 | 0.86-1.23 |
| Kuwabara, S 2024 | 1.09 | 0.91-1.31 |
| Marks, W. H 2019 | 1.02 | 0.83-1.05 |
| Garnier, A 2023 | 1.04 | 0.85-1.27 |

RR: relative risk; CI: confidence interval.


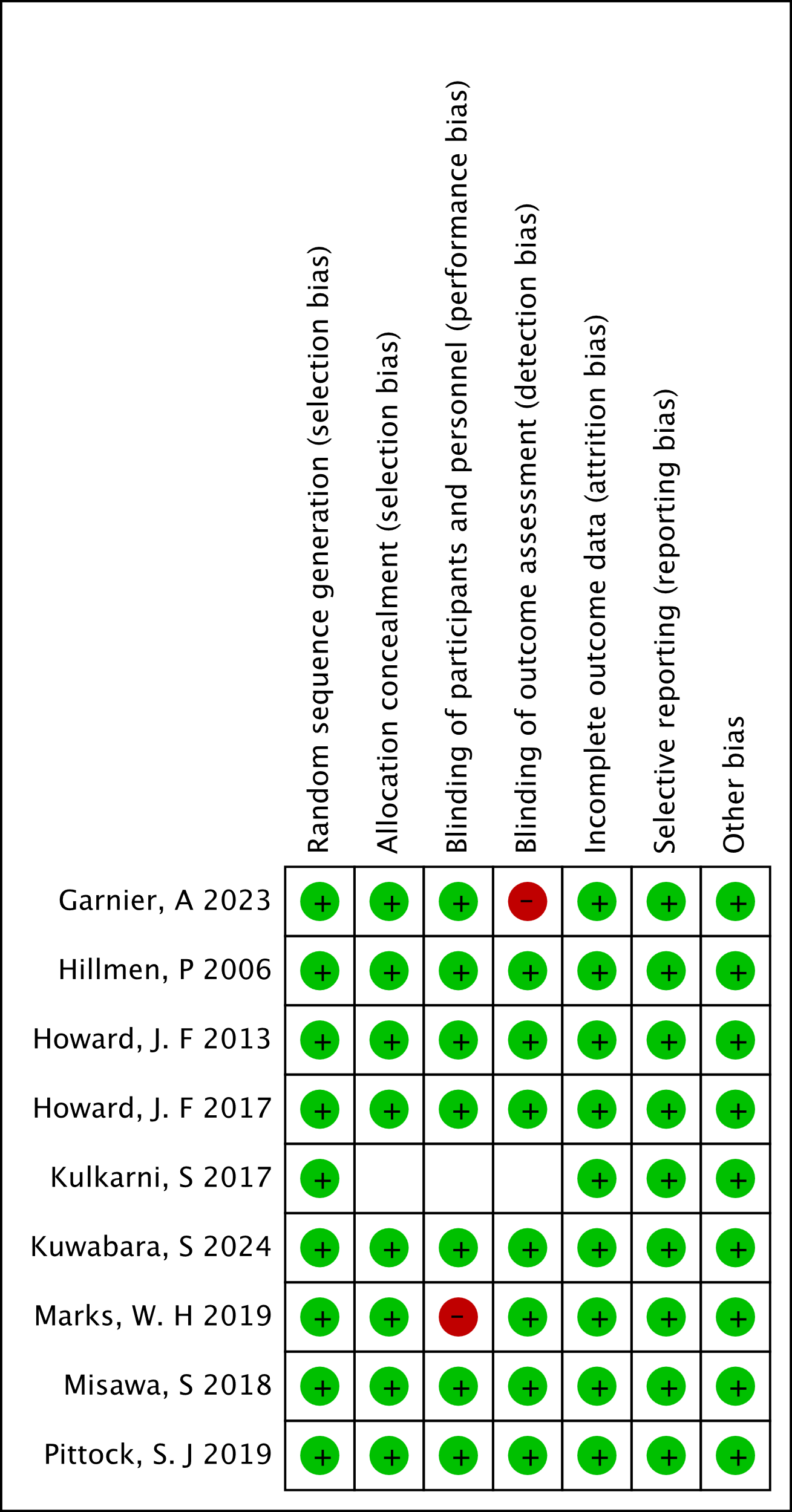


**Supplementary FIGURE S1** Risk of bias summary: review of authors’ judgments about each risk of bias item for each included study.


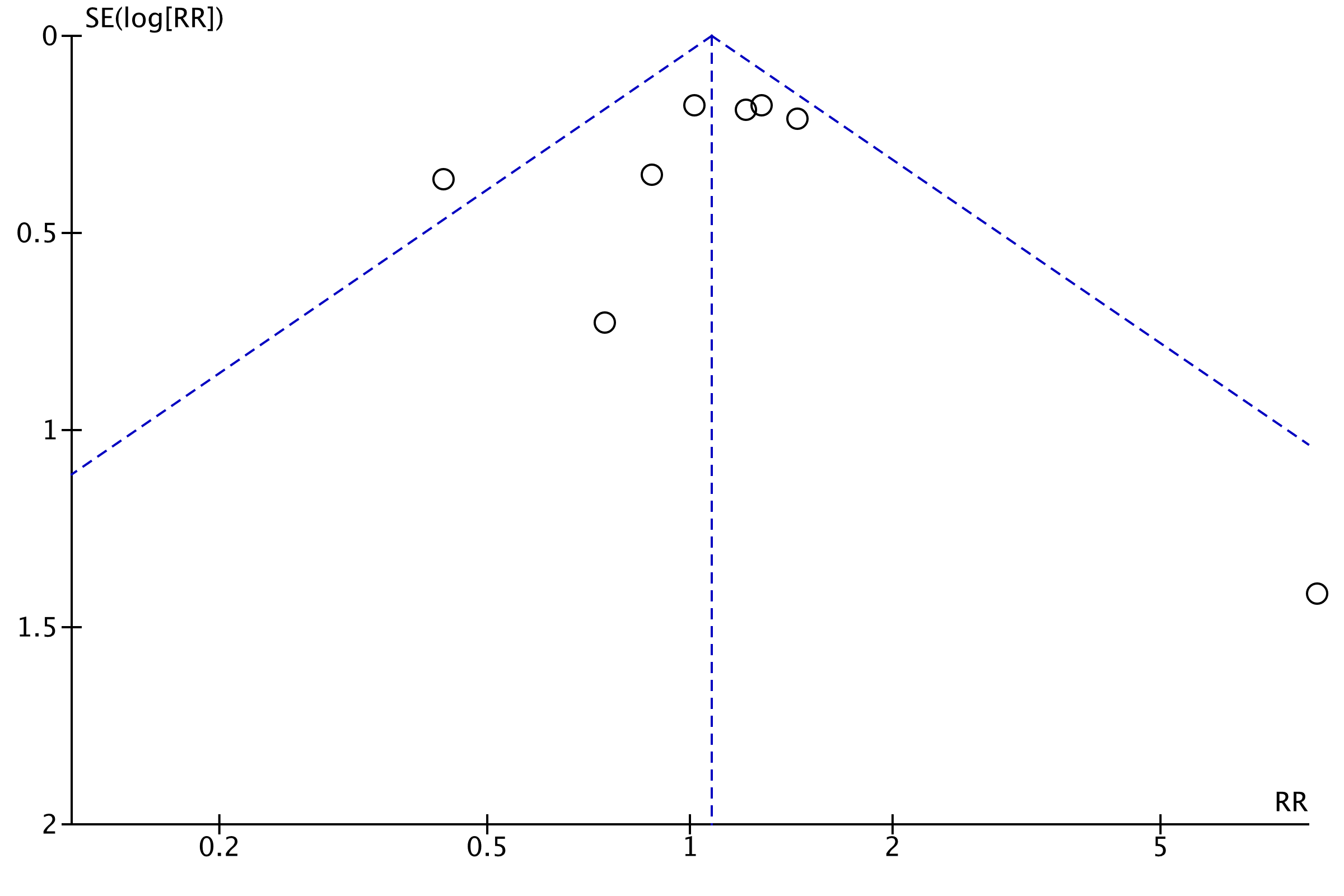


**Supplementary FIGURE S2** Funnel plot of the overall risk of infection associated with Eculizumab.


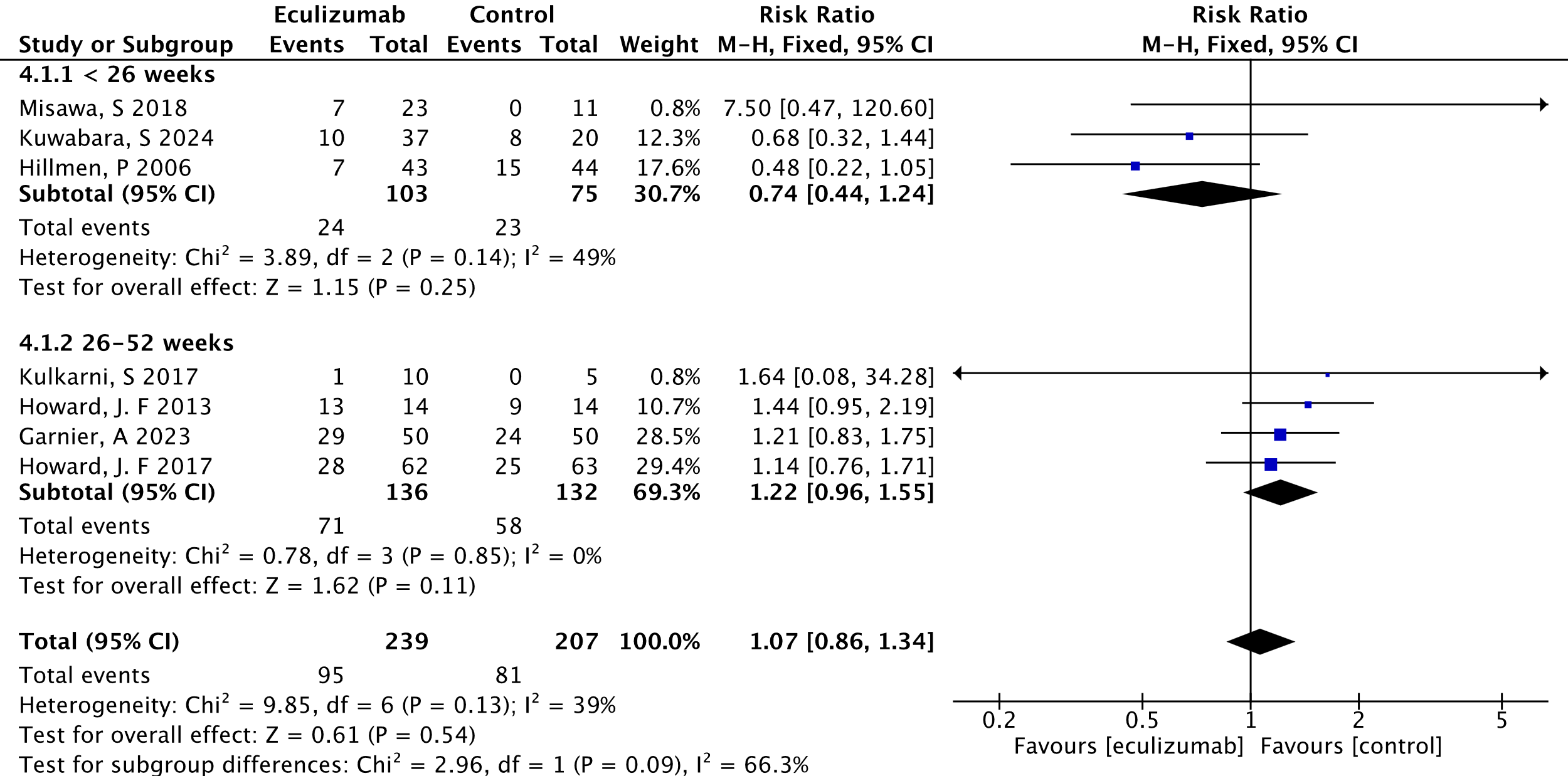


**Supplementary FIGURE S3** Forest plot with meta-analysis of the overall risk of general infection by different follow-ups associated with Eculizumab.


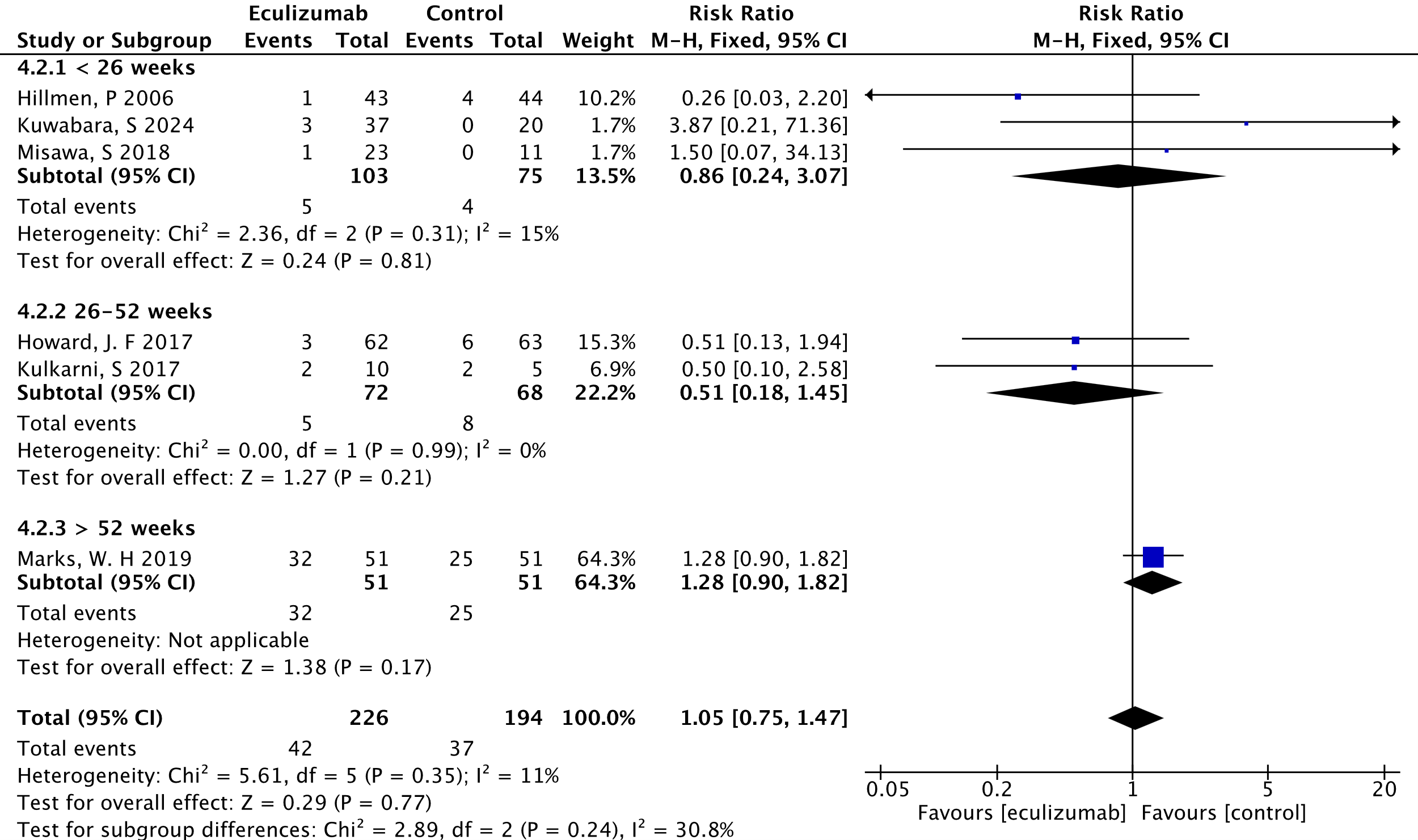


**Supplementary FIGURE S4** Forest plot with meta-analysis of the overall risk of severe infection by different follow-ups associated with Eculizumab.


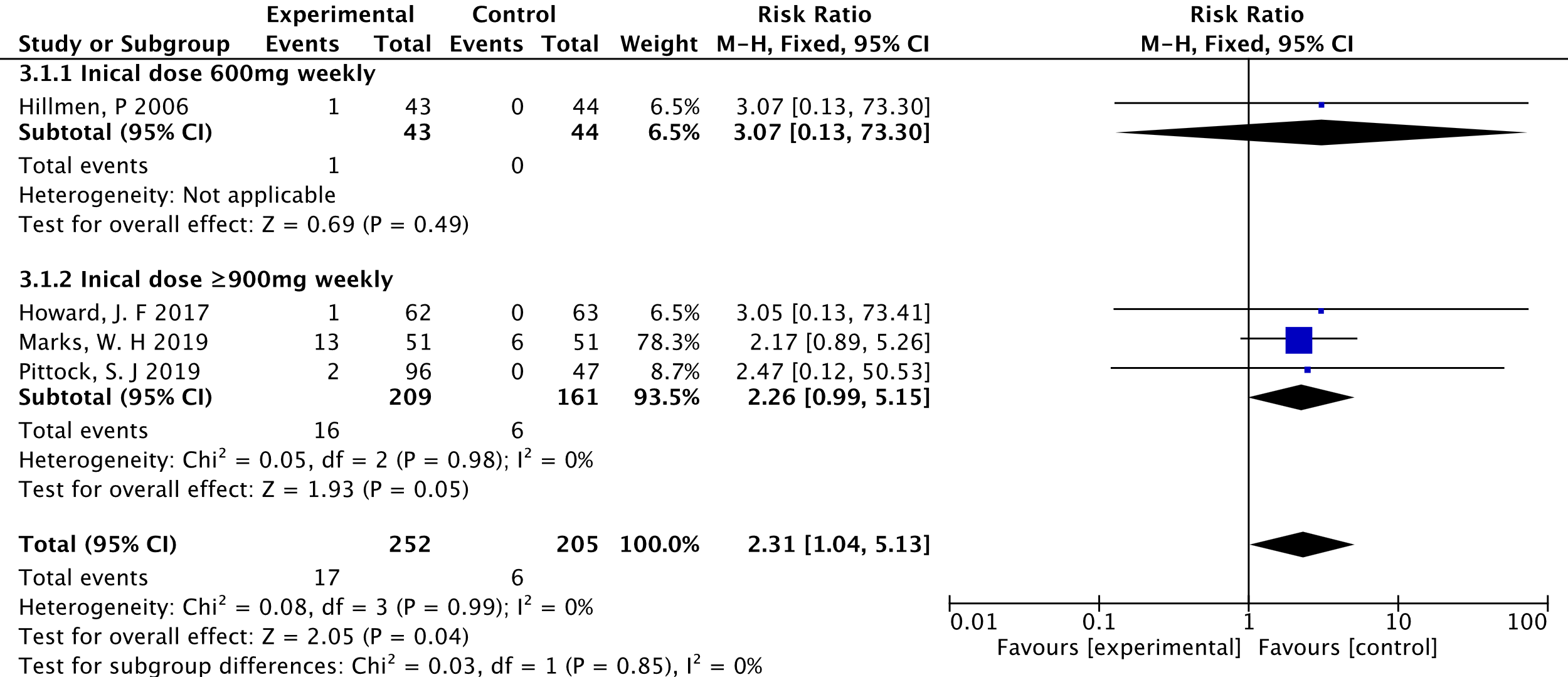


**Supplementary FIGURE S5** Forest plot with meta-analysis of the risk of severe bacteraemia by different initial dose associated with Eculizumab.
